# Supplementary material for: Direct observation of coordinated DNA movements on the nucleosome during chromatin remodelling
Source: Nat Commun. 2019 Apr 12;10:1720. doi: 10.1038/s41467-019-09657-1 (PMC6461674; doi:10.1038/s41467-019-09657-1)
Supplement: Supplementary file 1 — Supplementary Information [file 41467_2019_9657_MOESM1_ESM.pdf]

Supplementary Information

Direct observation of coordinated DNA movements on the nucleosome during chromatin remodelling

Anton Sabantsev[1], Robert F. Levendosky[2], Xiaowei Zhuang[3,4,5], Gregory D. Bowman[2], and Sebastian Deindl[1]

[1]Department of Cell and Molecular Biology, Science for Life Laboratory, Uppsala University, SE-75237 Uppsala, Sweden. [2]T.C. Jenkins Department of Biophysics, Johns Hopkins University, Baltimore, MD 21218, USA. [3]Howard Hughes Medical Institute, [4]Department of Chemistry and Chemical Biology, [5]Department of Physics, Harvard University, Cambridge, MA 02138, USA.

These authors contributed equally: Anton Sabantsev, Robert F. Levendosky

Correspondence and requests for materials should be addressed to S.D. (email: sebastian.deindl@icm.uu.se) or to G.D.B. (email: gdbowman@jhu.edu)

Supplementary Note 1. Correction of three-colour smFRET traces.

Fluorescence emission time traces were corrected to account for the direct excitation of Cy5 and of Alexa750 (direct excitation of Alexa750 by the 532 nm laser was negligible), as well as for the bleedthrough from the Cy3 into the Cy5 channel and that from Cy5 into the Alexa750 channel. Cy3 and Alexa750 signals were scaled to correct for the differences in quantum yields and detection efficiencies between the three dyes.

637 nm excitation case:

$$I_{Cy5}^{Corr} = \frac{I_{Cy5}^{Raw}}{(1 - Cy5Leak)}$$

$$I_{A750}^{Corr} = \frac{\frac{I_{A750}^{Raw} - Cy5Leak * I_{Cy5}^{Corr}}{A750Scale} - A750Dir * I_{Cy5}^{Corr}}{1 + A750Dir}$$

$$F_{Cy5-A750} = \frac{I_{A750}^{Corr}}{\left(I_{Cy5}^{Corr} + I_{A750}^{Corr}\right)} \quad ,$$

where I_{Cy5}^{Raw}, I_{A750}^{Raw}, I_{Cy5}^{Corr} and I_{A750}^{Corr} are raw and corrected fluorescence intensities of Cy5 and A750 upon excitation with the 637 nm laser; $Cy5Leak$ (=0.0432) is the correction coefficient for the leakage of Cy5 fluorescence into the A750 channel; $A750Scale$ (=0.398) is the correction coefficient that accounts for the difference in quantum yield and registration efficiency between A750 and Cy5; $A750Dir$ (=0.1085) is the correction coefficient for the direct excitation of A750 with the 637 nm laser; and $F_{Cy5-A750}$ is FRET between Cy5 and A750.

532 nm excitation case:

$$I_{Cy3}^{Int} = \frac{I_{Cy3}^{Raw}}{(1 - Cy3Leak)}$$

$$I_{Cy5}^{Int} = \frac{I_{Cy5}^{Raw} - I_{Cy3}^{Int} * Cy3Leak}{(1 - Cy5Leak)}$$

$$I_{A750}^{Int} = \frac{I_{A750}^{Raw} - I_{Cy5}^{Int} * Cy5Leak}{A750Scale}$$

$$I_{Cy3}^{Corr} = \frac{I_{Cy3}^{Int}}{Cy3Scale}$$

$$I_{Total} = \frac{I_{Cy3}^{Corr} + I_{Cy5}^{Int} + I_{A750}^{Int}}{1 + Cy5Dir}$$

$$I_{Cy5}^{Corr} = I_{Cy5}^{Int} - Cy5Dir * \left(1 - F_{Cy5-A750}\right) * I_{Total}$$

$$I_{A750}^{Corr} = I_{A750}^{Int} - Cy5Dir * F_{Cy5-A750} * I_{Total}$$

$$F_{From\ Cy3} = 1 - \frac{I_{Cy3}^{Corr}}{I_{Total}} \quad ,$$

where I_{Cy3}^{Raw}, I_{Cy5}^{Raw}, I_{A750}^{Raw}, I_{Cy3}^{Int}, I_{Cy5}^{Int}, I_{A750}^{Int}, I_{Cy3}^{Corr}, I_{Cy5}^{Corr} and I_{A750}^{Corr} are raw, intermediate and corrected fluorescence intensities of Cy3, Cy5, and A750 upon excitation with the 532 nm laser; $Cy3Leak$ (=0.0996) and $Cy5Leak$ (=0.0432) are correction coefficients for the leakage of Cy3 fluorescence into the Cy5 channel and of Cy5 fluorescence into the A750 channel respectively; $Cy3Scale$ (=0.92) and $A750Scale$ (=0.398) are correction coefficients that accounts for the difference in quantum yield and registration efficiency between Cy3 and Cy5 or

A750 and Cy5 respectively; $Cy5Dir$ (=0.103) is the correction coefficient for the direct excitation of Cy5 with the 532 nm laser; I_{Total} is the total fluorescence intensity of Cy3, Cy5 and A750 after all the corrections; and $F_{From\ Cy3}$ is the total FRET originating from Cy3. Leakage of Cy3 fluorescence into the A750 channel as well as the direct excitation of A750 with the 532 nm laser were neglected.

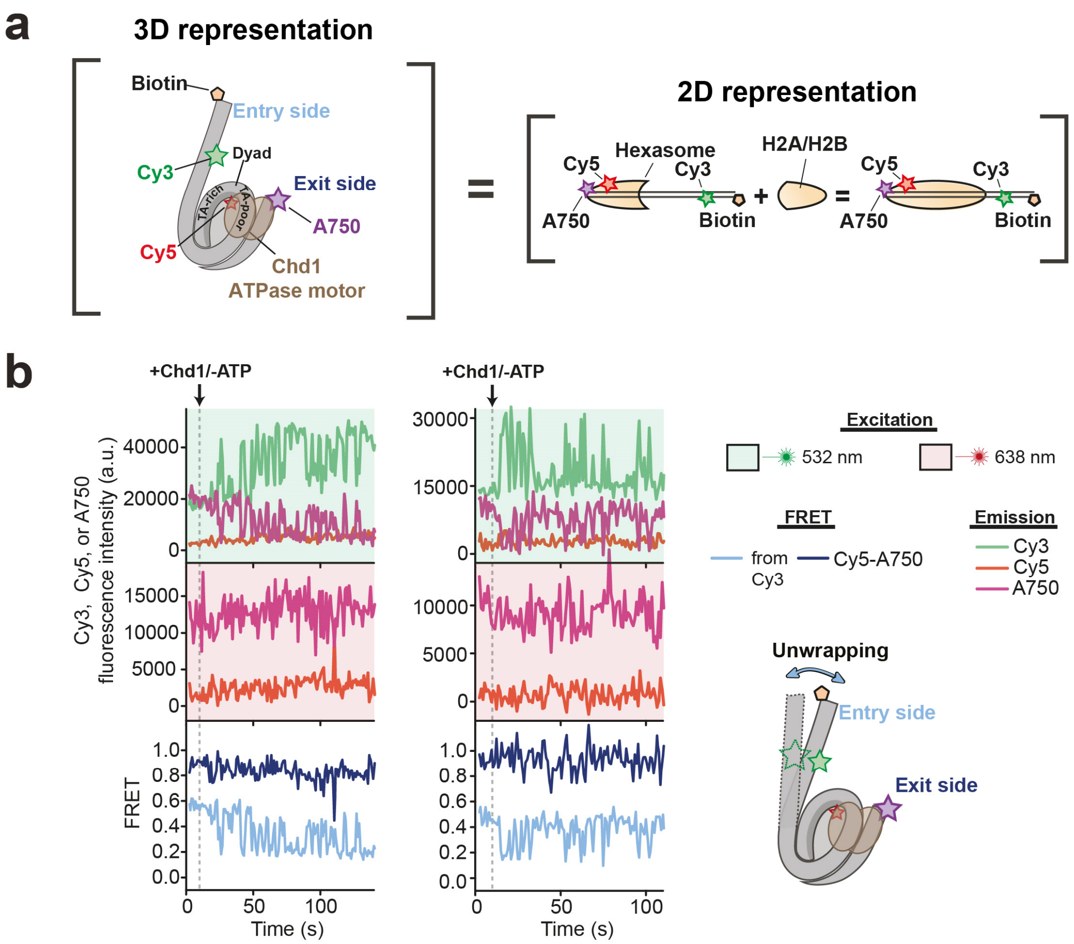

Supplementary Figure 1. Three-colour smFRET nucleosomes with external fluorophore positions are affected by Chd1-induced DNA unwrapping. (**a**) Two equivalent, schematic representations of the three-colour labelling scheme with external fluorophore positions. The right side explicitly shows assembly of nucleosomes from hexasomes and H2A/H2B dimers. (**b**) Representative Cy3 (green), Cy5 (red), and Alexa750 (purple) fluorescence and FRET (originating from Cy3: light blue; Cy5-Alexa750: dark blue) time traces showing unwrapping of DNA from the edge of a nucleosome with external fluorophore positions (cartoon, bottom right) after addition of 300 nM Chd1 alone (no ATP) at time indicated with a dashed line. Time traces were recorded with alternating 532 nm and 638 nm laser excitation, as indicated by the areas shaded in green and red, respectively.

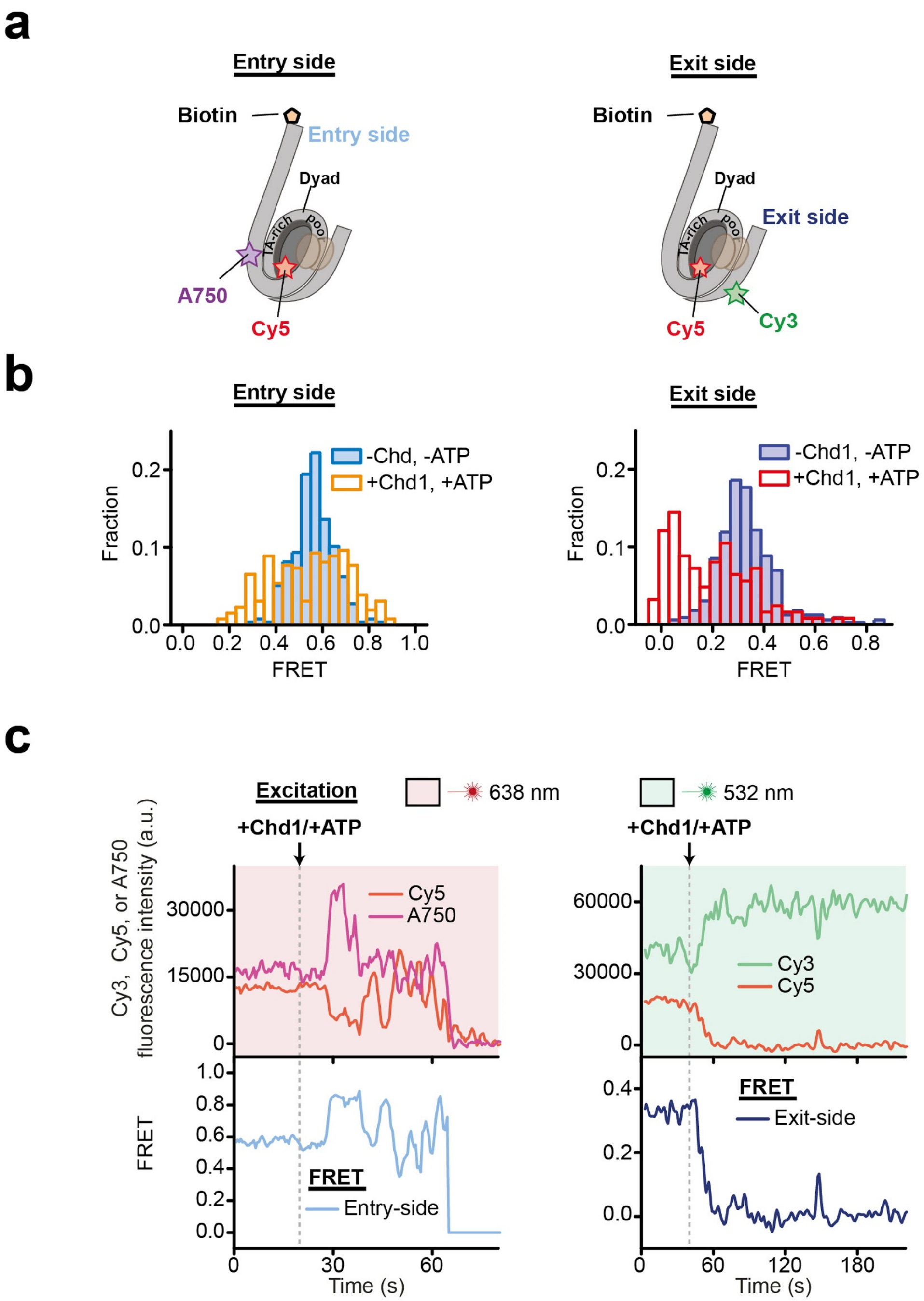

Supplementary Figure 2. Two-colour versions of the three-colour FRET nucleosomes exhibit initial FRET values and FRET changes during remodelling similar to those observed for the three-colour FRET nucleosomes. (**a**) Cartoons of two-colour versions of three-colour FRET nucleosomes lacking the exit-side Cy3 (left) or the entry-side A750 label (right). Cy3, Cy5, and Alexa750 are depicted by green, red, and purple stars, respectively. The labels on either side of the dyad indicate the TA-poor and TA-rich sides of the 601 sequence. (**b**) FRET histograms constructed from many entry (left) or exit (right) side two-colour FRET nucleosomes before (dark blue: exit side; light blue: entry side) and after (red: exit side; orange: entry side) the addition of 300 nM Chd1 with 1 mM ATP. (**c**) Representative Cy3 (green), Cy5 (red), Alexa750 (purple) fluorescence and FRET (light blue: entry side; dark blue: exit side) time traces showing sliding of a single entry (left) or exit (right) side two-colour FRET nucleosome after addition of 300 nM Chd1 and 1 mM ATP at the dashed line. All FRET histograms were constructed from >120 two-colour FRET nucleosomes. Source data are provided as a Source Data file.

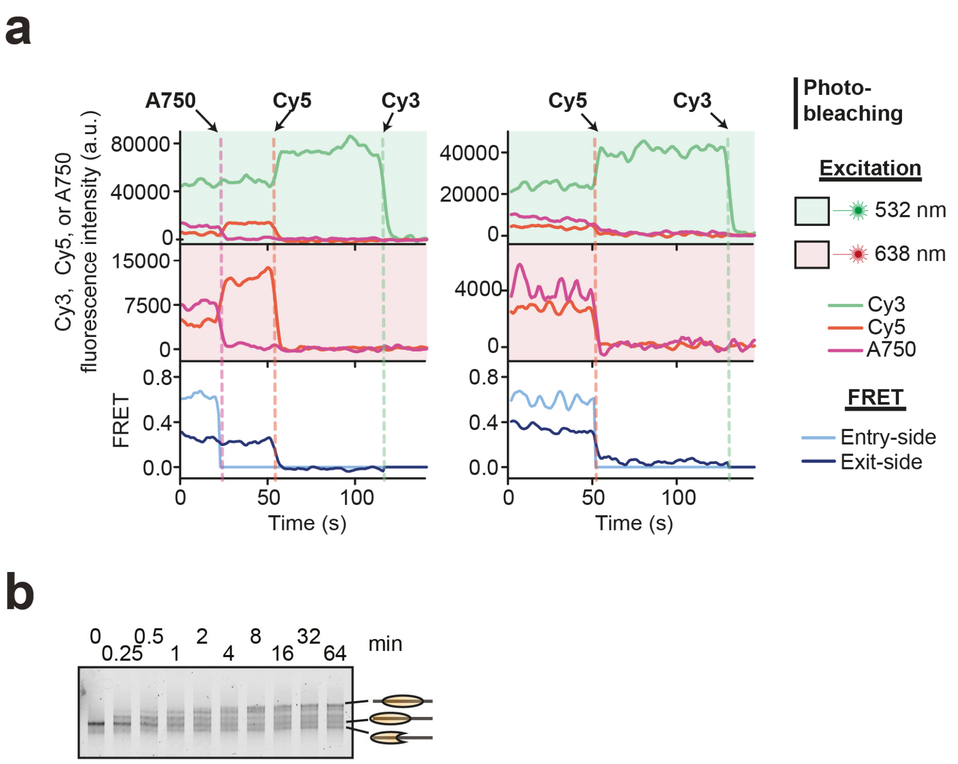

Supplementary Figure 3. Three-colour FRET nucleosomes display single photobleaching steps for Cy3, Cy5, and A750 and can be remodelled by Chd1. (**a**) Representative Cy3 (green), Cy5 (red), A750 (purple) fluorescence, entry-side FRET (light blue), and exit-side FRET (dark blue) time traces from single three-colour FRET nucleosomes showing single photobleaching steps as indicated by the dashed lines. Time traces were recorded with alternating 532 and 638 nm laser excitation, as indicated by the areas shaded in green and red, respectively. (**b**) Native gel electrophoresis of three-colour FRET nucleosomes (150 nM) before and after a 0.25, 0.5, 1, 2, 4, 8, 16, 32, and 64 min incubation with 50 nM Chd1 and 2.5 mM ATP. Source data are provided as a Source Data file.

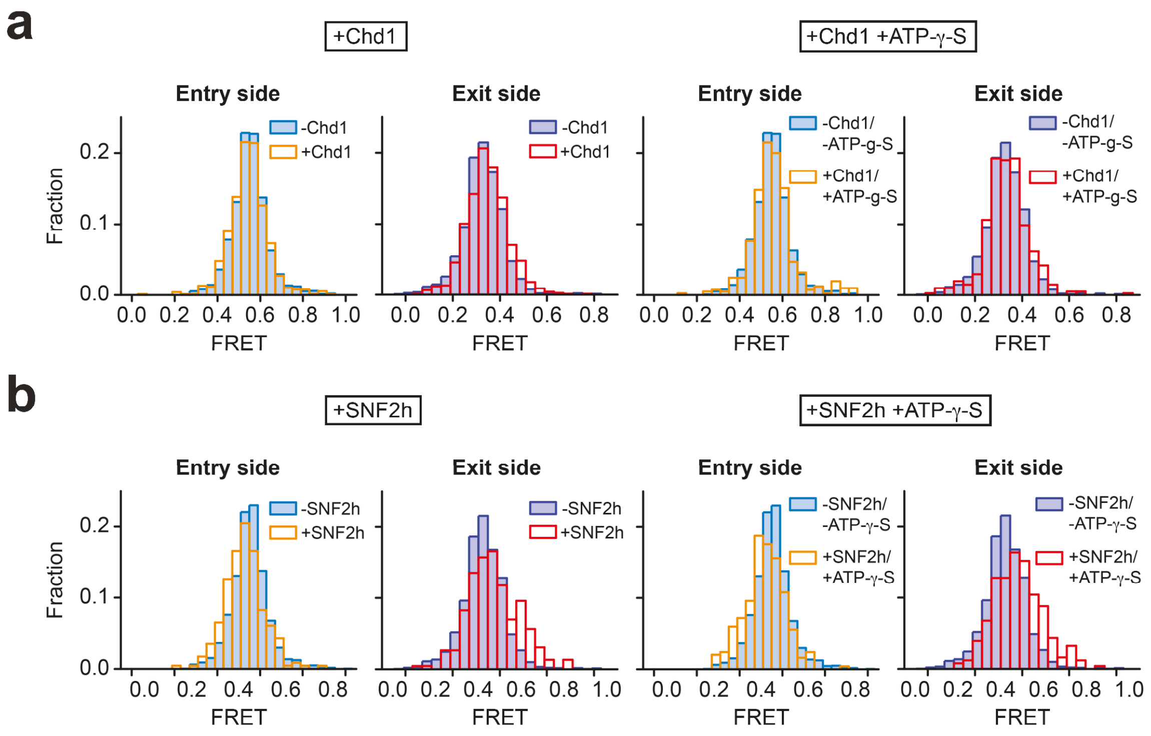

Supplementary Figure 4. Three-colour FRET nucleosomes with internal fluorophore positions do not show FRET fluctuations induced by Chd1 or SNF2h in the absence of ATP hydrolysis. Entry-side (left panels) and exit-side (right panels) FRET histograms before (dark blue: exit side; light blue: entry side) and after (red: exit side; orange: entry side) the addition of 300 nM Chd1 (**a**) or 1μM SNF2h (**b**) alone in the absence of a nucleotide (left) or with 1 mM ATP-γ-S (right). All FRET histograms were constructed from >200 (**b**) or >400 (**a**) nucleosomes. Source data are provided as a Source Data file.

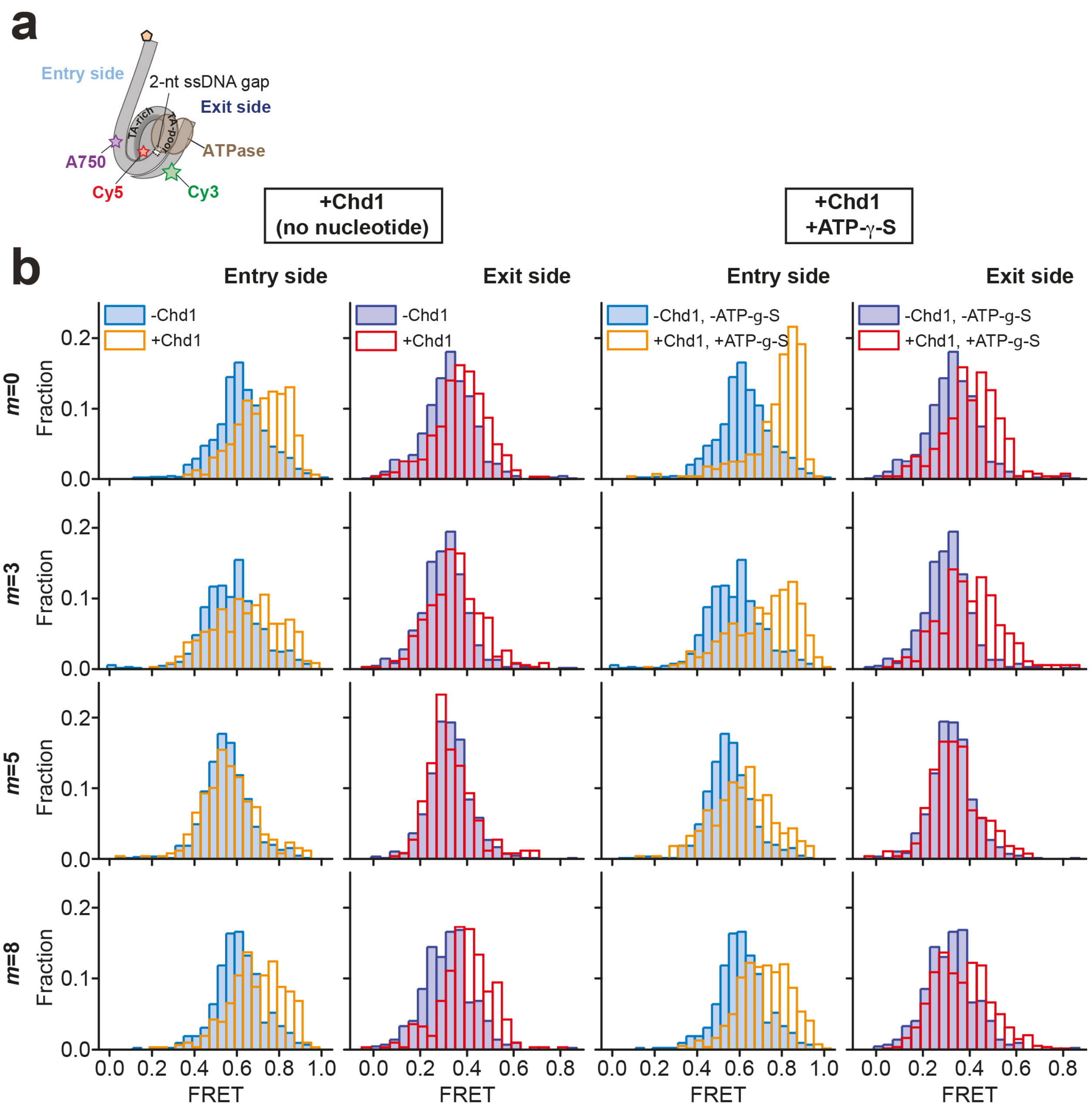

Supplementary Figure 5. Three-colour FRET imaging does not permit an unambiguous observation of DNA movements for gapped nucleosomes. (**a**) Cartoon representation of a three-colour FRET nucleosome with a 2-nt ssDNA gap near the entry SHL2. The labels on either side of the dyad indicate the TA-poor and TA-rich sides of the 601 sequence. (**b**) Entry-side (left) and exit-side (right) FRET histograms constructed from gapped nucleosomes before (dark blue: exit side; light blue: entry side) and after (red: exit side; orange: entry side) the addition of 300 nM Chd1 alone in the absence of ATP (left side) or with 1 mM ATP-γ-S (right side). All FRET histograms were constructed from >250 nucleosomes. Source data are provided as a Source Data file.

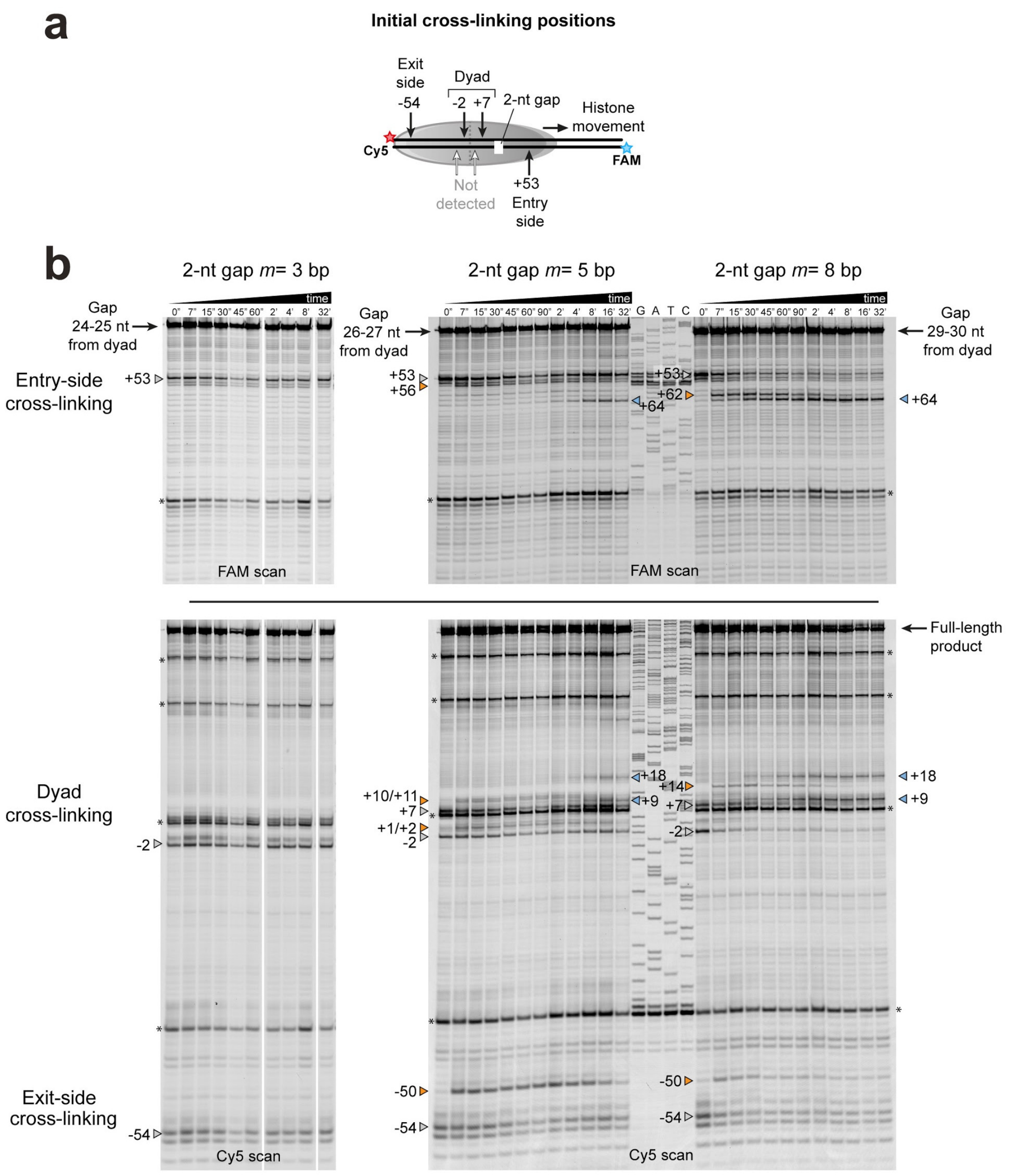

Supplementary Figure 6. Urea denaturing gel from histone-DNA cross-linking experiments, using nucleosomes with single-stranded DNA (ssDNA) gaps close to SHL2. (**a**) Schematic depicting site-specific DNA cross-linking expected for H2B(S53C) and H3(M120C) (black and white vertical arrows). Cross-links from H2B(S53C) report on both the entry and exit sides, cross-linking the top strand -54 nt from the dyad on the exit side (dark blue), and the bottom strand +53 nt from the dyad on the entry side (light blue). Cross-links from H3(M120C) report on the dyad, with top strand cross-linking initially occurring -2 and +7 nt from the dyad. Due to the 2-nt gap on the bottom strand (white box), cross-links made by H3(M120C) to the bottom strand are not observed. (**b**) Raw gel data used for generating plots in Fig. 3. A time-course of histone-DNA cross-linking was carried out for 150 nM nucleosome, 200 nM Chd1, and 1 mM ATP, using *m*=3, *m*= 5 and *m*= 8 bp nucleosomes. After processing, cross-linking reactions were resolved on urea polyacrylamide gels. Top image is a FAM scan, showing entry side cross-links, and bottom image is a Cy5 scan, showing exit side and dyad cross-links. colouring scheme matches that of Fig. 3, with gray indicating starting positions, orange indicating products that appear quickly, at the first time point, and blue indicating products that appear more slowly. Asterisks indicate intermediate DNA products not fully ligated during DNA preparations. Source data are provided as a Source Data file.

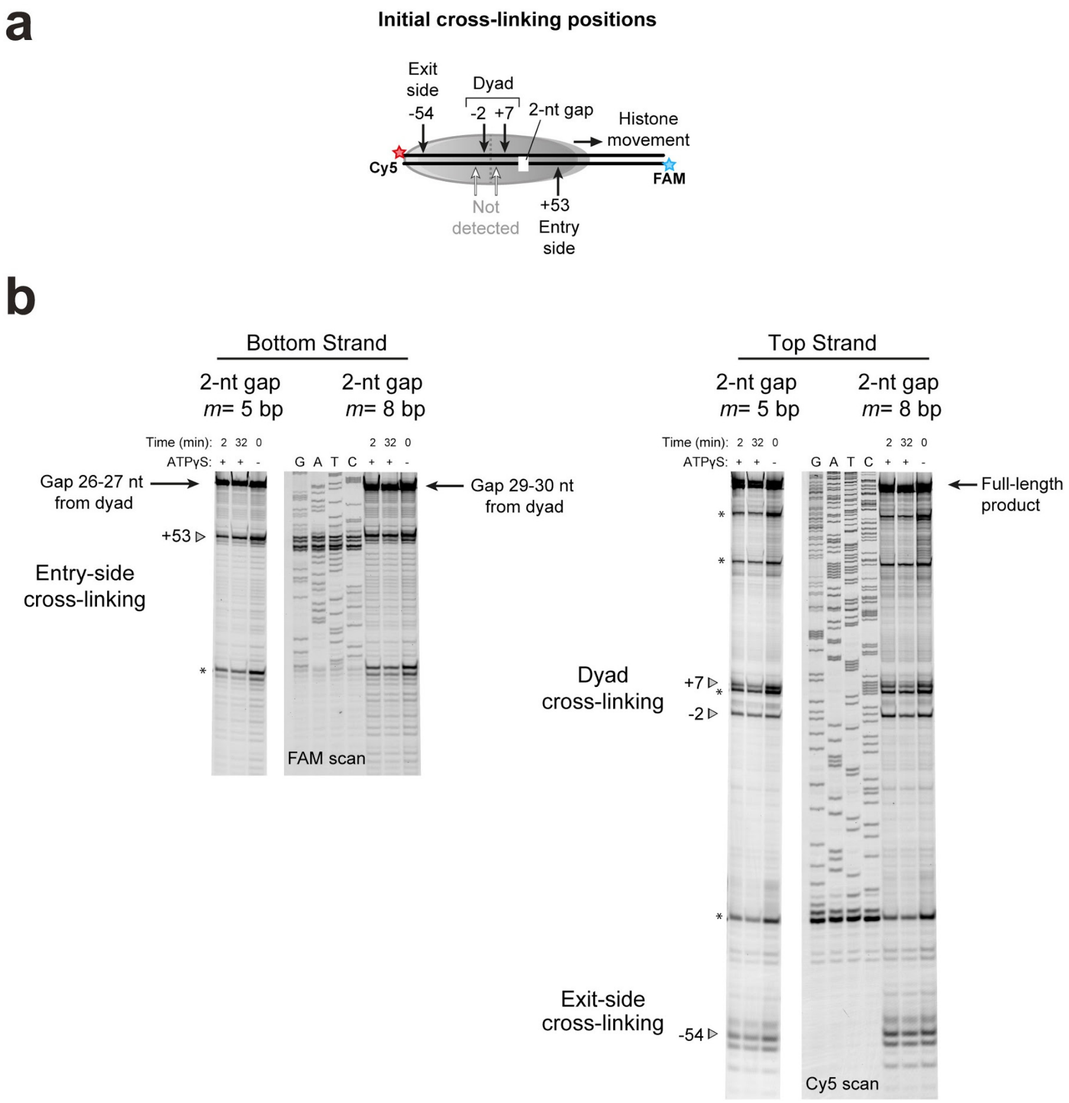

Supplementary Figure 7. Site-specific crosslinking of gapped nucleosomes is not altered by Chd1 in the presence of ATP-γ-S. (**a**) Schematic depicting the initial crosslinking positions of histones to DNA. (**b**) Urea denaturing gels of remodeling reactions containing 150 nM *m*= 5 or *m*= 8 nucleosomes and 200 nM Chd1 with and without 1 mM ATP-γ-S. Reactions with ATP-γ-S were incubated at room temperature for 2' and 32' before UV crosslinking. Source data are provided as a Source Data file.

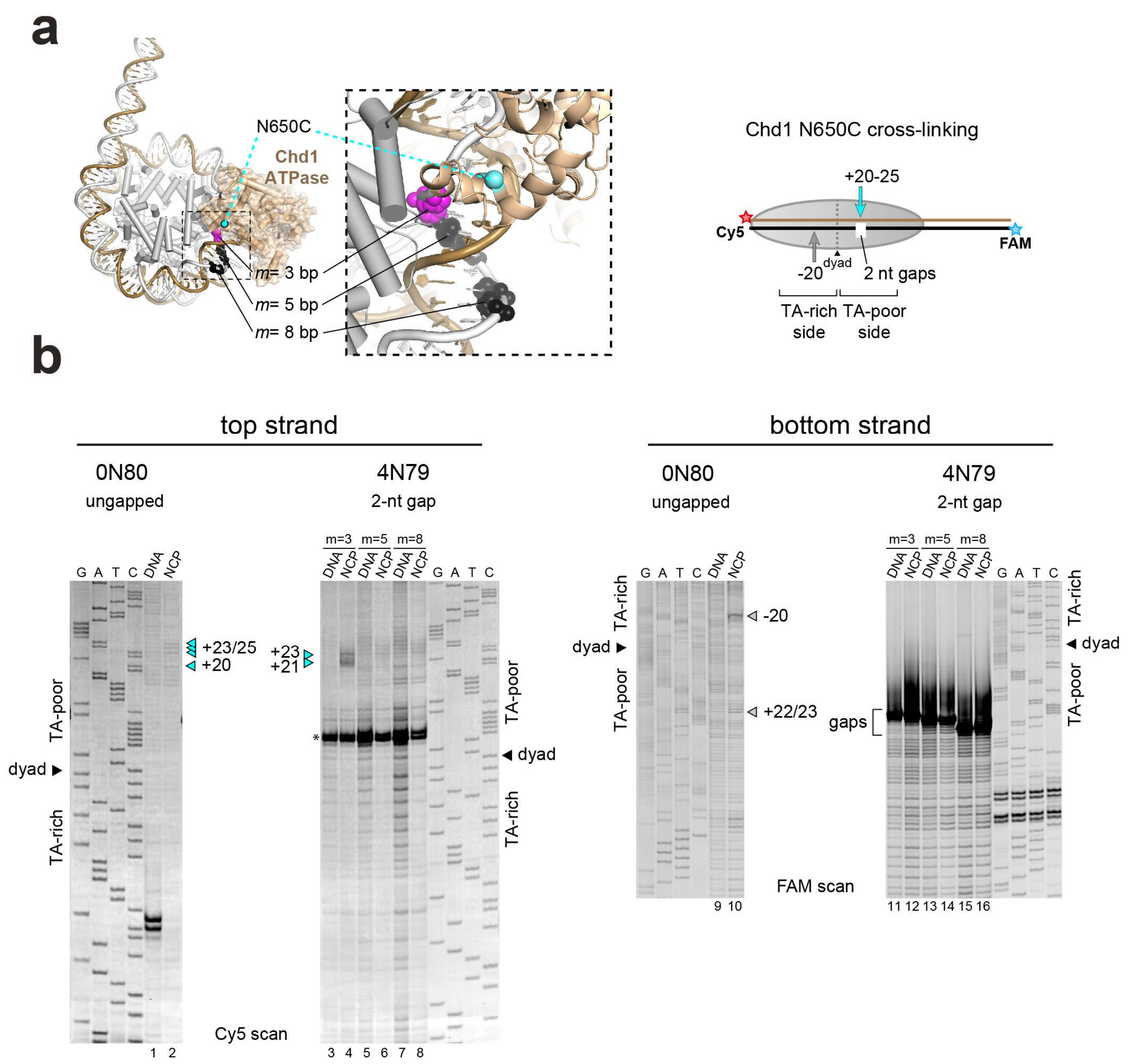

Supplementary Figure 8. Crosslinking of the Chd1 ATPase motor to nucleosomal DNA shows a different behavior with *m*= 3 bp gapped nucleosomes. (**a**) A model (left) of the Chd1-nucleosome structure (pdb code: 6FTX [ref. 1]), showing only the ATPase motor of Chd1 and indicating position 650 where a cysteine was introduced for site-specific cross-linking (N650C, cyan). The locations of 2 nt gaps are shown as spheres (magenta for *m*= 3, black for *m*= 5, 8), with the DNA strand opposite the gapped strand colored bronze. Diagram (right) shows a 2D representation, highlighting the location of cross-links on the top strand, which are opposite the gap. (**b**) Chd1 cross-linked more strongly to the *m*= 3 bp gapped nucleosome, opposite the 2 nt gap. Shown are denaturing urea gels separating nicked DNA fragments from Chd1 crosslinking reactions using ungapped (0N80) and gapped (4N79) nucleosomes. Note the stronger products for *m*= 3 bp nucleosomes in lane 4 (cyan arrowheads). Reactions contained 150 nM free DNA or nucleosome (NCP), 450 nM Chd1 (N650C), and 2 mM ADP·BeF_3^- (2 mM ADP, 15 mM NaF, 3 mM $BeCl_2$, and 6 mM $MgCl_2$). Reactions were carried out similarly to cross-linking for H2B(S53C) and H3(M120C), with Chd1(N650C) labeled with azido phenacyl bromide. The cysteine-free, N650C Chd1 variant was previously described[2]. Source data are provided as a Source Data file.

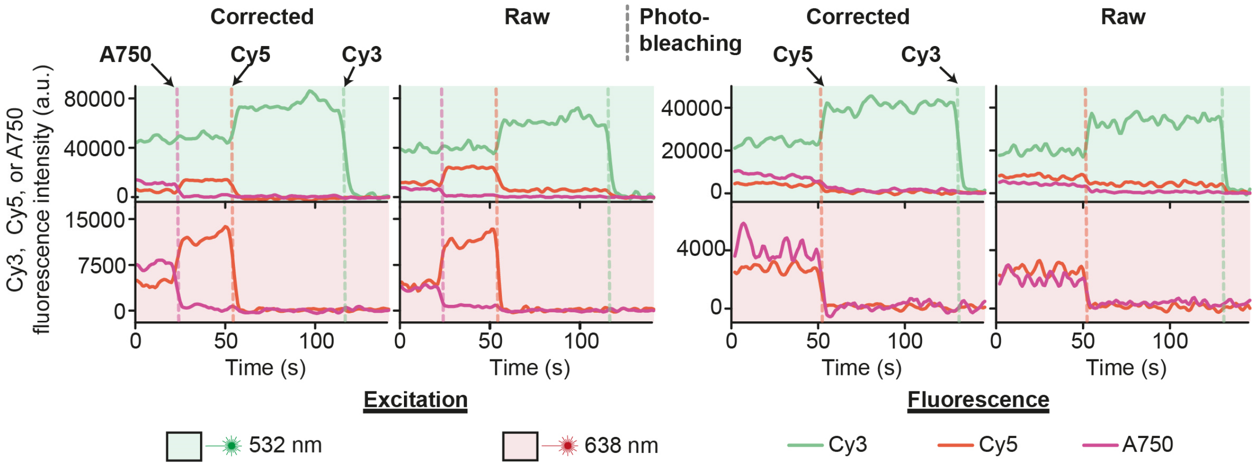

Supplementary Figure 9. Examples of three-colour smFRET traces before and after correction. Representative Cy3 (green), Cy5 (red), A750 (purple) fluorescence time traces from single three-colour FRET nucleosomes before and after corrections for Cy3 and Cy5 bleedthrough, direct excitation of Cy5 and A750 by 532 nm and 637 nm lasers, respectively, as well as for differences in fluorescence quantum yields and detection efficiencies between Cy3, Cy5 and A750.

Supplementary References

1. Sundaramoorthy, R. *et al.* Structure of the chromatin remodelling enzyme Chd1 bound to a ubiquitinylated nucleosome. *Elife* **7** (2018).

2. Nodelman, I. M. *et al.* Interdomain Communication of the Chd1 Chromatin Remodeler across the DNA Gyres of the Nucleosome. *Mol Cell* **65**, 447-459 (2017).

<table>
<tr><td colspan="2">Supplementary Table 1: DNA sequences of mononucleosomes described in this manuscript. The TA-rich and TA-poor halves of the 601 positioning sequence are shaded in dark and light grey, respectively.</td></tr>
<tr><td colspan="2">Legend: 601 = “TA-rich side - dyad - TA-poor side”, 2-nt ssDNA gap = “--”</td></tr>
<tr><td>Top strand</td><td>Bottom strand</td></tr>
<tr><td colspan="2">1) Three-colour FRET construct with labels outside the nucleosome positioning sequence. Oligonucleotides: F1_A750, F2, F3, F4, F5, R1, R2, R3_iCy3, R4</td></tr>
<tr><td>5’-Biotin□GGTACCCGTAGATCCTCTAGAGTGGGAGCTCGGAACACTATCCGACTGG
CACCGGCAAGGTCGCTGTT□iCy3□AATACATGCACAGGATGTATATATCTGACACGTG
CCTGGAGACTAGGGAGTAATCCCCTTGGCGGTTAAAACGCGGGGGACAGCGCGTACGTGC
GTTTAAGCGGTGCTAGAGCTGTCTACGACCAATTGAGCGGCCTGCAGACCGGGATTCTCC
AGGGC□3’</td><td>5’-Alexa750-GCCCTGGAGAATCCCGGTCTGCAGGCCGCTCAATTGGTCGTAGACAG
CTCTAGCACCGCTTAAACGCACGTACGCGCTGTCCCCCGCGTTTTAACCGCCAAGGGGA
TTACTCCCTAGTCTCCAGGCACGTGTCAGATATATACATCCTGTGCATGTATTGAACAG
CGACCTTGCCGGTGCCAGTCGGATAGTGTTCCGAGCTCCCACTCTAGAGGATCTACGGG
TACC-3’</td></tr>
<tr><td colspan="2">2) Three-colour FRET construct with labels inside the nucleosome positioning sequence. Oligonucleotides: F1_new, F2, F3_A750, F4, F5_new, R1_iCy3, R2_new, R3_new, R4_new</td></tr>
<tr><td>5’-Biotin-GGTACCCGTAGATCCTCTAGAGTGGGAGCTCGGAACACTATCCGACTGGC
ACCGGCAAGGTCGCTGTTCAATACATGCACAGGATGTATATATCTGACACGTGCCTGGAG
ACTAGGGAGTAATCCCCTTGGCGGTTAAAACGCGGGGGACAGCGCGTACGTGCGTTTAAG
CGGTGCTAGAGCTGTCTACGACCAATTGAG-iCy3-GGCCTGCAGACCGGGATTCTCCAG
GGC-3’</td><td>5’-GCCCTGGAGAATCCCGGTCTGCAGGCCACTCAATTGGTCGTAGACAGCTCTAGCAC
CGCTTAAACGCACGTACGCGCTGTCCCCCGCGTTTTAACCGCCAAGGGGATTACTCCCT
AGTCTCCAGGCACGTG-T-Alexa750-CAGATATATACATCCTGTGCATGTATTGAAC
AGCGACCTTGCCGGTGCCAGTCGGATAGTGTTCCGAGCTCCCACTCTAGAGGATCTACG
GGTACC-3’</td></tr>
<tr><td colspan="2">3) Three-colour FRET construct with internal labels and the inverted orientation of the 601 sequence, “601-flip”. Oligonucleotides: FF1, FF2, FF3_A750, FF4, F5_new, FR1_iCy3, FR2, FR3, R4_new</td></tr>
<tr><td>5’-Biotin-GGTACCCGTAGATCCTCTAGAGTGGGAGCTCGGAACACTATCCGACTGGC
ACCGGCAAGGTCGCTGTTCAATACATGCCTGGAGAATCCCGGTCTGAAGGCCGCTCAATT
GGTCGTAGACAGCTCTAGCACCGCTTAAACGCACGTACGCGCTGTCCCCCGCGTTTTAAC
CGCCAAGGGGATTACTTCCTAGTCTCCAGG-iCy3-ACGTGTCAGATATATACATCCTGT
GGC-3’</td><td>5’-GCCACAGGATGTATATATCTGACACGTACCTGGAGACTAGGAAGTAATCCCCTTGG
CGGTTAAAACGCGGGGGACAGCGCGTACGTGCGTTTAAGCGGTGCTAGAGCTGTCTACG
ACCAATTGAGCGGCCT-T-Alexa750-CAGACCGGGATTCTCCAGGCATGTATTGAAC
AGCGACCTTGCCGGTGCCAGTCGGATAGTGTTCCGAGCTCCCACTCTAGAGGATCTACG
GGTACC-3’</td></tr>
<tr><td colspan="2">4) Exit-side two-colour version of the three-colour FRET construct with labels inside the nucleosome positioning sequence. Oligonucleotides: F1_new, F2, F3, F4, F5_new, R1_iCy3, R2_new, R3_new, R4_new</td></tr>
<tr><td>5’-Biotin-GGTACCCGTAGATCCTCTAGAGTGGGAGCTCGGAACACTATCCGACTGGC
ACCGGCAAGGTCGCTGTTCAATACATGCACAGGATGTATATATCTGACACGTGCCTGGAG
ACTAGGGAGTAATCCCCTTGGCGGTTAAAACGCGGGGGACAGCGCGTACGTGCGTTTAAG
CGGTGCTAGAGCTGTCTACGACCAATTGAG-iCy3-GGCCTGCAGACCGGGATTCTCCAG
GGC-3’</td><td>5’-GCCCTGGAGAATCCCGGTCTGCAGGCCACTCAATTGGTCGTAGACAGCTCTAGCAC
CGCTTAAACGCACGTACGCGCTGTCCCCCGCGTTTTAACCGCCAAGGGGATTACTCCCT
AGTCTCCAGGCACGTGTCAGATATATACATCCTGTGCATGTATTGAACAGCGACCTTGC
CGGTGCCAGTCGGATAGTGTTCCGAGCTCCCACTCTAGAGGATCTACGGGTACC-3’</td></tr>
<tr><td colspan="2">5) Entry-side two-colour version of the three-colour FRET construct with labels inside the nucleosome positioning sequence. Oligonucleotides: F1_new, F2, F3_A750, F4, F5_new, R1_new, R2_new, R3_new, R4_new.</td></tr>
<tr><td>5’-Biotin-GGTACCCGTAGATCCTCTAGAGTGGGAGCTCGGAACACTATCCGACTGGC
ACCGGCAAGGTCGCTGTTCAATACATGCACAGGATGTATATATCTGACACGTGCCTGGAG
ACTAGGGAGTAATCCCCTTGGCGGTTAAAACGCGGGGGACAGCGCGTACGTGCGTTTAAG
CGGTGCTAGAGCTGTCTACGACCAATTGAGTGGCCTGCAGACCGGGATTCTCCAGGGC-3
’</td><td>5’-GCCCTGGAGAATCCCGGTCTGCAGGCCACTCAATTGGTCGTAGACAGCTCTAGCAC
CGCTTAAACGCACGTACGCGCTGTCCCCCGCGTTTTAACCGCCAAGGGGATTACTCCCT
AGTCTCCAGGCACGTG-T-Alexa750-CAGATATATACATCCTGTGCATGTATTGAAC
AGCGACCTTGCCGGTGCCAGTCGGATAGTGTTCCGAGCTCCCACTCTAGAGGATCTACG
GGTACC-3’</td></tr>
<tr><td colspan="2">6) Three-colour FRET construct with labels inside the nucleosome positioning sequence and an ssDNA gap in the m= 0 position. Oligonucleotides: F1_new, F2, F3_A750, F4, F5_new, R1_iCy3, R2_Gap0, R3_Gap0, R4_new</td></tr>
<tr><td>5’-Biotin-GGTACCCGTAGATCCTCTAGAGTGGGAGCTCGGAACACTATCCGACTGGC
ACCGGCAAGGTCGCTGTTCAATACATGCACAGGATGTATATATCTGACACGTGCCTGGAG
ACTAGGGAGTAATCCCCTT--CGGTTAAAACGCGGGGGACAGCGCGTACGTGCGTTTAAG
CGGTGCTAGAGCTGTCTACGACCAATTGAG-iCy3-GGCCTGCAGACCGGGATTCTCCAG
GGC-3’</td><td>5’-GCCCTGGAGAATCCCGGTCTGCAGGCCACTCAATTGGTCGTAGACAGCTCTAGCAC
CGCTTAAACGCACGTACGCGCTGTCCCCCGCGTTTTAACCGCCAAGGGGATTACTCCCT
AGTCTCCAGGCACGTG-T-Alexa750-CAGATATATACATCCTGTGCATGTATTGAAC
AGCGACCTTGCCGGTGCCAGTCGGATAGTGTTCCGAGCTCCCACTCTAGAGGATCTACG
GGTACC-3’</td></tr>
<tr><td colspan="2">7) Three-colour FRET construct with labels inside the nucleosome positioning sequence and an ssDNA gap in the m= 3 position. Oligonucleotides: F1_new, F2, F3_A750, F4, F5_new, R1_iCy3, R2_Gap3, R3_Gap3, R4_new</td></tr>
<tr><td>5’-Biotin-GGTACCCGTAGATCCTCTAGAGTGGGAGCTCGGAACACTATCCGACTGGC
ACCGGCAAGGTCGCTGTTCAATACATGCACAGGATGTATATATCTGACACGTGCCTGGAG
ACTAGGGAGTAATCCC--TGGCGGTTAAAACGCGGGGGACAGCGCGTACGTGCGTTTAAG
CGGTGCTAGAGCTGTCTACGACCAATTGAG-iCy3-GGCCTGCAGACCGGGATTCTCCAG
GGC-3’</td><td>5’-GCCCTGGAGAATCCCGGTCTGCAGGCCACTCAATTGGTCGTAGACAGCTCTAGCAC
CGCTTAAACGCACGTACGCGCTGTCCCCCGCGTTTTAACCGCCAAGGGGATTACTCCCT
AGTCTCCAGGCACGTG-T-Alexa750-CAGATATATACATCCTGTGCATGTATTGAAC
AGCGACCTTGCCGGTGCCAGTCGGATAGTGTTCCGAGCTCCCACTCTAGAGGATCTACG
GGTACC-3’</td></tr>
<tr><td colspan="2">8) Three-colour FRET construct with labels inside the nucleosome positioning sequence and an ssDNA gap in the m= 5 position. Oligonucleotides: F1_new, F2, F3_A750, F4, F5_new, R1_iCy3, R2_Gap5, R3_Gap5, R4_new</td></tr>
<tr><td>5’-Biotin-GGTACCCGTAGATCCTCTAGAGTGGGAGCTCGGAACACTATCCGACTGGC
ACCGGCAAGGTCGCTGTTCAATACATGCACAGGATGTATATATCTGACACGTGCCTGGAG
ACTAGGGAGTAATC--CTTGGCGGTTAAAACGCGGGGGACAGCGCGTACGTGCGTTTAAG
CGGTGCTAGAGCTGTCTACGACCAATTGAG-iCy3-GGCCTGCAGACCGGGATTCTCCAG
GGC-3’</td><td>5’-GCCCTGGAGAATCCCGGTCTGCAGGCCACTCAATTGGTCGTAGACAGCTCTAGCAC
CGCTTAAACGCACGTACGCGCTGTCCCCCGCGTTTTAACCGCCAAGGGGATTACTCCCT
AGTCTCCAGGCACGTG-T-Alexa750-CAGATATATACATCCTGTGCATGTATTGAAC
AGCGACCTTGCCGGTGCCAGTCGGATAGTGTTCCGAGCTCCCACTCTAGAGGATCTACG
GGTACC-3’</td></tr>
</table>

<table>
<tr><td colspan="2">9) Three-colour FRET construct with labels inside the nucleosome positioning sequence and an ssDNA gap in the m= 8 position. Oligonucleotides: F1_new, F2, F3_A750, F4, F5_new, R1_iCy3, R2_Gap8, R3_Gap8, R4_new</td></tr>
<tr><td>5’-Biotin-GGTACCCGTAGATCCTCTAGAGTGGGAGCTCGGAACACTATCCGACTGGC
ACCGGCAAGGTCGCTGTTCAATACATGCACAGGATGTATATATCTGACACGTGCCTGGAG
ACTAGGGAGTA--CCCCTTGGCGGTTAAAACGCGGGGGACAGCGCGTACGTGCGTTTAAG
CGGTGCTAGAGCTGTCTACGACCAATTGAG-iCy3-GGCCTGCAGACCGGGATTCTCCAG
GGC-3’</td><td>5’-GCCCTGGAGAATCCCGGTCTGCAGGCCACTCAATTGGTCGTAGACAGCTCTAGCAC
CGCTTAAACGCACGTACGCGCTGTCCCCCGCGTTTTAACCGCCAAGGGGATTACTCCCT
AGTCTCCAGGCACGTG-T-Alexa750-CAGATATATACATCCTGTGCATGTATTGAAC
AGCGACCTTGCCGGTGCCAGTCGGATAGTGTTCCGAGCTCCCACTCTAGAGGATCTACG
GGTACC-3’</td></tr>
<tr><td colspan="2">10) Cross-linking construct with an ssDNA gap in the m= 3 position. Oligonucleotides: F1_Cy5, F2, F3, F4, F5_new, R1_new, R2_Gap3, R3_Gap3, R4_FAM</td></tr>
<tr><td>5’-FAM-GGTACCCGTAGATCCTCTAGAGTGGGAGCTCGGAACACTATCCGACTGGCACC
GGCAAGGTCGCTGTTCAATACATGCACAGGATGTATATATCTGACACGTGCCTGGAGACT
AGGGAGTAATCCC--TGGCGGTTAAAACGCGGGGGACAGCGCGTACGTGCGTTTAAGCGG
TGCTAGAGCTGTCTACGACCAATTGAGTGGCCTGCAGACCGGGATTCTCCAGGGC-3’</td><td>5’-Cy5-GCCCTGGAGAATCCCGGTCTGCAGGCCACTCAATTGGTCGTAGACAGCTCTA
GCACCGCTTAAACGCACGTACGCGCTGTCCCCCGCGTTTTAACCGCCAAGGGGATTACT
CCCTAGTCTCCAGGCACGTGTCAGATATATACATCCTGTGCATGTATTGAACAGCGACC
TTGCCGGTGCCAGTCGGATAGTGTTCCGAGCTCCCACTCTAGAGGATCTACGGGTACC-
3’</td></tr>
<tr><td colspan="2">11) Cross-linking construct with an ssDNA gap in the m= 5 position. Oligonucleotides: F1_Cy5, F2, F3, F4, F5_new, R1_new, R2_Gap5, R3_Gap5, R4_FAM</td></tr>
<tr><td>5’-FAM-GGTACCCGTAGATCCTCTAGAGTGGGAGCTCGGAACACTATCCGACTGGCACC
GGCAAGGTCGCTGTTCAATACATGCACAGGATGTATATATCTGACACGTGCCTGGAGACT
AGGGAGTAATC--CTTGGCGGTTAAAACGCGGGGGACAGCGCGTACGTGCGTTTAAGCGG
TGCTAGAGCTGTCTACGACCAATTGAGTGGCCTGCAGACCGGGATTCTCCAGGGC-3’</td><td>5’-Cy5-GCCCTGGAGAATCCCGGTCTGCAGGCCACTCAATTGGTCGTAGACAGCTCTA
GCACCGCTTAAACGCACGTACGCGCTGTCCCCCGCGTTTTAACCGCCAAGGGGATTACT
CCCTAGTCTCCAGGCACGTGTCAGATATATACATCCTGTGCATGTATTGAACAGCGACC
TTGCCGGTGCCAGTCGGATAGTGTTCCGAGCTCCCACTCTAGAGGATCTACGGGTACC-
3’</td></tr>
<tr><td colspan="2">12) Cross-linking construct with an ssDNA gap in the m= 8 position. Oligonucleotides: F1_Cy5, F2, F3, F4, F5_new, R1_new, R2_Gap8, R3_Gap8, R4_FAM</td></tr>
<tr><td>5’-FAM-GGTACCCGTAGATCCTCTAGAGTGGGAGCTCGGAACACTATCCGACTGGCACC
GGCAAGGTCGCTGTTCAATACATGCACAGGATGTATATATCTGACACGTGCCTGGAGACT
AGGGAGTA--CCCCTTGGCGGTTAAAACGCGGGGGACAGCGCGTACGTGCGTTTAAGCGG
TGCTAGAGCTGTCTACGA CCAATTGAGTGGCCTGCAGACCGGGATTCTCCAGGGC-3’</td><td>5’-Cy5-GCCCTGGAGAATCCCGGTCTGCAGGCCACTCAATTGGTCGTAGACAGCTCTA
GCACCGCTTAAACGCACGTACGCGCTGTCCCCCGCGTTTTAACCGCCAAGGGGATTACT
CCCTAGTCTCCAGGCACGTGTCAGATATATACATCCTGTGCATGTATTGAACAGCGACC
TTGCCGGTGCCAGTCGGATAGTGTTCCGAGCTCCCACTCTAGAGGATCTACGGGTACC-
3’</td></tr>
</table>

Supplementary Table 2: Oligonucleotide sequences (5' to 3') used for generating nucleosomal DNA constructs shown in Supplementary Table 1.

Name	Sequence
F1_A750	/5Alex750N/GCCCTGGAGAATCCCGGTCTGCAGGCCGCTCAATT
F1_new	GCCCTGGAGAATCCCGGTCTGCAGGCCACTCAATT
F1_Cy5	/5Cy5/GCCCTGGAGAATCCCGGTCTGCAGGCCACTCAATT
F2	/5phos/GGTCGTAGACAGCTCTAGCACCGCTTAAACGCACGTACGCGCTGTC
F3	/5Phos/CCCCGCGTTTTAACCGCCAAGGGGATTACTCCCTAGTCTCCAGGCACGTGTCAGATATATAC
F3_A750	/5phos/CCCCGCGTTTTAACCGCCAAGGGGATTACTCCCTAGTCTCCAGGCACGTG/iAlex750N/CAGATATATAC
F4	/5phos/ATCCTGTGCATGTATTGAACAGCGACCTTGCCGGTGCCAGTCGGAT
F5	/5phos/AGTGTTCCGAGCTCCCACTCTAGAGGATCCCCGGGTACC
F5_new	/5phos/AGTGTTCCGAGCTCCCACTCTAGAGGATCTACGGGTACC
R1	/5Phos/CGGTGCTAGAGCTGTCTACGACCAATTGAGCGGCCTGCAGACCGGGATTCTCCAGGGC
R1_new	/5phos/CGGTGCTAGAGCTGTCTACGACCAATTGAGTGGCCTGCAGACCGGGATTCTCCAGGGC
R1_iCy3	/5phos/CGGTGCTAGAGCTGTCTACGACCAATTGAG/iCy3/GGCCTGCAGACCGGGATTCTCCAGGGC
R2	/5Phos/GACTAGGGAGTAATCCCCTTGGCGGTTAAAACGCGGGGGACAGCGCGTACGTGCGTTTAAG
R2_new	/5phos/TAATCCCCTTGGCGGTTAAAACGCGGGGGACAGCGCGTACGTGCGTTTAAG
R2_Gap0	CGGTTAAAACGCGGGGGACAGCGCGTACGTGCGTTTAAG
R2_Gap3	TGGCGGTTAAAACGCGGGGGACAGCGCGTACGTGCGTTTAAG
R2_Gap5	CTTGGCGGTTAAAACGCGGGGGACAGCGCGTACGTGCGTTTAAG
R2_Gap8	CCCCTTGGCGGTTAAAACGCGGGGGACAGCGCGTACGTGCGTTTAAG
R3_iCy3	/5Phos/GTCGCTGTT/iCy3/AATACATGCACAGGATGTATATATCTGACACGTGCCTGGA
R3_new	/5phos/GTCGCTGTTCAATACATGCACAGGATGTATATATCTGACACGTGCCTGGAGACTAGGGAG
R3_Gap0	/5phos/GTCGCTGTTCAATACATGCACAGGATGTATATATCTGACACGTGCCTGGAGACTAGGGAGTAATCCCCTT
R3_Gap3	/5phos/GTCGCTGTTCAATACATGCACAGGATGTATATATCTGACACGTGCCTGGAGACTAGGGAGTAATCCC
R3_Gap5	/5phos/GTCGCTGTTCAATACATGCACAGGATGTATATATCTGACACGTGCCTGGAGACTAGGGAGTAATC
R3_Gap8	/5phos/GTCGCTGTTCAATACATGCACAGGATGTATATATCTGACACGTGCCTGGAGACTAGGGAGTA
R4	/5BiotinTEG/GGTACCCGGGGATCCTCTAGAGTGGGAGCTCGGAACACTATCCGACTGGCACCGGCAAG
R4_new	/5BiotinTEG/GGTACCCGTAGATCCTCTAGAGTGGGAGCTCGGAACACTATCCGACTGGCACCGGCAAG
R4_FAM	/56-FAM/AGTACCCGTAGATCCTCTAGAGTGGGAGCTCGGAACACTATCCGACTGGCACCGGCAAG
FF1	GCCACAGGATGTATATATCTGACACGTACCTGGAG
FF2	/5phos/ACTAGGAAGTAATCCCCTTGGCGGTTAAAACGCGGGGGACAGC
FF3_A750	/5phos/GCGTACGTGCGTTTAAGCGGTGCTAGAGCTGTCTACGACCAATTGAGCGGCCT/iAlex750N/CAGACCGGG
FF4	/5phos/ATTCTCCAGGCATGTATTGAACAGCGACCTTGCCGGTGCCAGTCGGAT
FR1_iCy3	/5phos/CGCCAAGGGGATTACTTCCTAGTCTCCAGG/iCy3/ACGTGTCAGATATATACATCCTGTGGC
FR2	/5phos/GGTCGTAGACAGCTCTAGCACCGCTTAAACGCACGTACGCGCTGTCCCCCGCGTTTTAAC
FR3	/5phos/GTCGCTGTTCAATACATGCCTGGAGAATCCCGGTCTGAAGGCCGCTCAATT
